# The Institutional Library

**Published:** 1919-01-04

**Authors:** 


### THE INSTITUTIONAL LIBRARY.

### THE FUTURE OF PHYSICAL TREATMENT.

ONE of the great physicians of the later Victorian epoch, addressing his pupils, told them that "Medicine is the metropolis of the kingdom of Knowledge, and we are the privileged denizens thereof." It is a fine and true metaphor, provided we can cast away what is so apt to become exclusive in our privilege and accept knowledge and opportunity for what they really are, entrusted to us for the service of our fellows.

The appearance of an admirable English translation of a new edition of Dr. Emil Kleen's book* reminds us that in Sweden and to a less extent in our own country a new "little medical profession" has grown up side by side with the old one. These are the "gymnasts" and masseurs, and the professors of physical treatment generally. The "Central Institute of Gymnastics" was founded in Stockholm in 1813. It has attempted to combine three very different things: military gymnastics, educational gymnastics, and the purely medical art of mechanical treatment for the cure of disease. The students and diplomates of this and similar institutes have for many years practised their art to a large extent independently of the medical profession, and often as the rivals and antagonists of qualified medical men. All thoughtful persons must agree with Dr. Kleen that this is a deplorable state of affairs. It has become essential that the study and teaching of physical treatment should find a place, he truly says, "within the medical world: otherwise it is like a separate society which breaks away and artificially separates itself and becomes hostile to the world to which it belongs."

It must, however, be conceded that the training institutes have been founded with the laudable object of protecting the public from the dangerous attentions of a great army of entirely ignorant and unqualified persons. If a "little medical profession," with its own authorisation, certificates, and diplomas, is now practically in a destructive rivalry with the doctors, the latter are historically themselves to blame, because the medical profession has unfortunately taken but little interest in physical remedies, and retired "according to plan" from a very valuable and fertile piece of territory. Many thinkers and teachers, medical and non-medical, have raised their voices in recent years in protest against this neglect of a wide field of medicine by medical men. To this protest Dr. Kleen adds the weight derived from his long experience and authority.

The present volume is the nearest approach we have met with to an adequate standard treatise on Mechanical Treatment for the use of medical men, and the enlightened practitioner will be well repaid by its perusal. Under the general term "Mechanical Treatment" must be included not only manipulation (or massage), but all kinds of curative exercises, with and without apparatus. The rather rigid system of Swedish medical gymnastics, including positions and movements, is described and illustrated by Dr. J. Arvedson. This chapter also includes a good and practical account of the most valuable forms of manipulation (massage). There is a grand distinction to be drawn between soothing or sedative movements and manipulations and those that are stimulant or tonic. It is the distinction between a touch and a blow. The former relieve pain, take away restlessness, and even counteract acute inflammation, both by direct and reflex actions, which are too little understood. But vigorous manipulations act as a nervous tonic, and also directly upon the tissues, by increasing the circulation. Simple general ideas like these, disentangled from polyglot jargon, lie at the root of physical treatment. If the practitioner can apprehend their real meaning he will find it easier to solve the difficult question that constantly confronts him—Does the patient need rest or does he need exercise, or, if both, in what proportion? The rest cure of Weir Mitchell and his followers stands at the one pole of treatment: ergotherapy and the Swedish gymnastic system stand at the other. But physical remedies, sedative and stimulant, have play all along the line. Dr. Kleen suggests that a clue may be found in "innervation." Gymnastics, in his view, are essentially tonic, and therefore, indicated in hypotonic disease (deficient innervation); whilst hypertonic disease (increased innervation) essentially requires rest. The matter is not so easily disposed of, and is one of those questions of great practical importance which call for further examination and research on physiological lines. In the meanwhile it is something to know that all gymnastics and active manipulations are injurious in acute heart affections and even neuroses of the heart, in all febrile and inflammatory diseases, diabetes, irritable neurasthenia, and high arterial tension. But all of these disorders are, however, benefited by the more sedative forms of physical treatment.

Dr. Patrick Haglund contributes a section dealing with the application of gymnastics to orthopædics, using the latter word in its pre-war significance as the "study of the deformities of the human body." Indeed, there is in the volume under review but little reference to war experience. Many of the general statements of this experienced surgeon find ample confirmation in the treatment of war-disabled men—for example, the following. The patient's own efforts in daily work very often give better results in improving the mobility and power of the limb than either gymnastics or massage. This

* *Massage and Medical Gymnastics.* By Dr. Emil A. G. KLEEN, with contributions by Dr. J. Arvedson. Dr. P. Haglund, and Dr. E. Zander. Translated by Dr. Mina Dobbie, with a Foreword by Major R. C. Elmslie, R.A.M.C.(T.). 618+vii. pages, with 182 Illustrations. (London: J. and A. Churchill. 1918. Price 21s.)

**The Future of Physical Treatment**—(*continued*).

general principle is of much importance, and should never be lost sight of in the after-care of wounded men. But to be of any service, curative work, like any other physical treatment, must be placed under competent medical supervision. Dr. Haglund would minimise fixation and immobility as far as possible. "Every stiff joint should be mobilised excepting the tuberculous," but so far from disparaging apparatus he finds that passive movements by pendulum apparatus are of the utmost value in joint affections. The reader will find a lucid explanation of the gymnastic treatment appropriate to "weight deformities," such as "flat-foot," and an excellent account of the use of manipulations in contusions and sprains.

The effect of movements and manipulations under water and after baths, of which great use has been made for wounded soldiers, although referred to, is insufficiently described, and little reference is made to the powerful reflex effects of manipulation and other surface treatments, whilst their direct action on the tissues is everywhere emphasised. Dr. Kleen shrewdly remarks that as a rule the specialist in mechanical treatment shows more enthusiasm than critical faculty. It might be retorted that persons more highly placed have as a rule shown much critical faculty and but little enthusiasm. Both are equally wanted in this branch of medicine. A horse cannot draw well without harness, but still less can harness move without a horse. On the whole it is better to have life without method than method without life.

The widespread use of physical remedies for the wounded in the Allied countries is a new phenomenon. It has brought to the notice of thoughtful surgeons perhaps more than to physicians the possibilities which belong to this branch of medicine. In a suggestive foreword to Dr. Kleen's book, Major Elmslie, speaking from the surgical standpoint, puts the matter in a nutshell. We must recover the lost ground. The elements of physical treatment must be included among the recognised medical studies, and the skilled operator must work with and for the medical profession. Unfortunately, what is potent for good may be capable of evil, and a good tool is dangerous in unskilled hands. How is it possible in the public interest that the best physical treatment can be made available, not only for sailors and soldiers, but for civilians? Everybody will admit that no methods of treatment ought to be practised upon the sick, the rational foundations of which are not well and truly laid, and instruction imparted to those who practise them. The problem of the future of physical treatment is ripe for solution. How, when, and where can the necessary facilities for study and teaching be provided? R. F. F.

---

**A Sister of a Certain Soldier.** By Dr. STEPHEN J. MAHER, Newhaven, Conn., U.S.A. (Press of the Tuttle, Morehouse and Taylor Company. Popular edition. Price 25 cents.)

THIS is an odd volume for an Englishman to read, and with the suddenness of a cinematograph it seems to plunge him into a melodramatic America. Really a tract on Burns's much abused theme "A man's a man, for a' that," it tells of a coloured girl in the States, a mulatto, who, enraged at the refusal of the theatres to admit her, of the shop assistants to serve her, of the white women to sit in her neighbourhood, determined to prevent her brother from enlisting. A doctor, to whom she unbosoms herself in a moment of excitement, suggests that she had better become a nurse. She goes home, studies the Bible, and decides to do so. An outbreak of sickness on a farm near by gives her a chance. The effect of the popular respect won for her by her efforts, for skilled help was lacking, was to persuade her to help to raise a company of coloured men for the war. Her hatred of the American flag turned with equal impulsiveness to "a wild love" for it. The moral is that good behaviour depends less on high character than on good treatment. She carries the company's flag in the procession to the station when the company leaves for the Front. She reduces the American women in the audience to tears by singing them a song. Soon afterwards, at the station itself, she has a heart attack, and dies with a broken prayer to "the flag" upon her lips. It seems an ideal theme for a cinematograph story. Englishmen would find it difficult to take it seriously did not other evidence of varied kinds support its claim to actuality, and were not these melodramatic happenings apparently of normal occurrence in the United States. In the old country we are not so hysterical on the subject of women, or flags, or publicity. But if America is an hysterical country, then such facts as we may glean of its hysteria are worth more attention than they generally receive.

**The War-Workers.** By E. M. DELAFIELD, Author of "Zella Sees Herself." (London: William Heinemann. Price 6s.)

A VERY clever sketch is afforded in these pages of the "driver" type of woman, familiar to most people engaged in voluntary labours. Charmian Vivian, Director of the Midland Supply Depôt, is a product of war conditions in her present stage of development, but she has existed ever since women began to take part in philanthropic work, and must have had her prototype in prehistoric days. The authoress adroitly presents her to the reader under the double aspect which she wears first to her overworked and partly hypnotised group of voluntary workers in the wretched hostel provided for them, and secondly, to her father, Sir Piers Vivian, breaking under the war strain, and to her much younger and keen-eyed mother. The hostel scenes are admirable. The little coterie of Miss Vivian's admirers and victims is depicted with delightful vivacity, and the arrival of Grace Jones, the new clerk, who declines to be hypnotised, but proves a splendid comrade in the hostel, throws a new and humorous light on the situation, and incidentally introduces a little mild love interest. The figure of Char Vivian is, and remains, the central study. The authoress has resisted the cheap temptation to regenerate her. These self-victimising drivers, whose lust is to dominate others, seldom alter in the smallest particular. But the book leaves her a little modified, accepting with less than former asperity the slavish devotion of her two satellites in their united efforts "to persuade Miss Vivian to her lunch every day." A capital book for the convalescent shelves of the hospital library.

(*Continued on page* 298.)

**The Institutional Library**—(*continued from p. 286*).

**Forty New Poems.** By William H. Davies. (London: A. C. Fifield, 13 Clifford's Inn, E.C. 4. 1918. Price 4s. net.)

Beautifully printed on excellent paper by a publisher with a proved eye for the form of a book, the latest volume of the famous Super-Tramp is exactly like its predecessors. No sincerer compliment could be given, for variety is a symptom of incomplete achievement: "the *artist* revolves in a cycle of masterpieces." Admirers of Burne-Jones never weary of meeting the same face not merely in every picture, but in every person in the same picture. At the height of artistic achievement monotony is a virtue, not a defect. It was characteristic of Leonardo da Vinci, of Wordsworth at his best, and, to come nearer home, of John Clare and William Barnes, both poets of the "rural pen" like Mr. Davies. He is the Peter Pan of Parnassus; the infantile lover of "woolly sheep," of "soft rain," of "nipple buds," and of the butterfly who

"doth ope and close
His wings, as babies work their toes."

This perfect innocence of the eye, even success has not spoiled. And if the greater psychological disturbance of success could not spoil it, the disturbance of a European war to a poet who mislaid his foot on his trampings was necessarily a minor (psychological) upheaval. The reference to the war in "My Old Acquaintance" relieves us of any anxiety. The Old Acquaintance, with her toothless gums, philosophises on the war as follows:

For me, I cannot relish food, or sleep
Till God sees fit to hold the Kaiser fast,
Stabbed, shot, or hanged—and his black soul
Sent into hell; to bubble, burn and squeal;
Think of the price of fish—and look at bacon."

It is perhaps necessary to remind modern people that a man's duty, alike in war and in peace, consists in doing that which he can do best, not necessarily that which the well-stored mind of his excitable old uncle may deem, from the arm-chair in the club-window, to be best for him. May it not have been, that loss of a foot, a Providential warning that Mr. Davies' duty was to march, not in line, but in metre? May not his fiery moment of testing have been the moment of popular success (he is the purest of our singers), no less severe for *his* soul than the moment when the whistle blows for the men with a pair of feet to "go over"? The decision, at all events, lay not in our hands. It becomes us, therefore, to accept it according to the measure of our humility. The Marthas of this world are as necessary as they are numerous: all honour to their devotion; but we have high authority for saying that the Marys have been given, by the Divine Prerogative, the better part, which, as Mr. Davies shows most exquisitely in his latest volume, shall not be taken away from them.

Four shillings for forty-two poems is less per poem than the present price of a newspaper. If we do not grudge that for a sheet destined in a few short hours to cloak our bread or light our fire, shall we grudge it for a handful of writings which contain such a message to the world as this?

"Stung by a spiteful wasp,
I let him go life-free.
That proved the difference
In him and me.

"For had I killed my foe,
It had proved me at once
The stronger wasp, and no
More difference."

This, please note, is not an argument against killing wasps. It is a familiar instance of the fact that the destruction of a wasp can be best accomplished without striking moral attitudes over its dead body. Mr. Davies' first editions are now treasured by connoisseurs, and even booksellers. The purchase of "Forty New Poems" is a safe investment, whatever may happen after the war.

**Gymnastic Treatment for Joint and Muscle Disabilities.** By Brevet Col. H. E. Deane, R.A.M.C. (London: Henry Frowde, Oxford University Press; Hodder and Stoughton, Warwick Square, E.C.)

Colonel Deane has explained in this little book how by the use of simple gymnastic exercises and by easy organised games function may be restored to joints and muscles disabled by wounds, nerve injuries, and injuries of other kinds. As he writes in his book, every such case is not suited to treatment by gymnastics, but far more are than is commonly supposed. He especially recommends trial in that class of case so common now that has resisted massage and treatment by passive movements for a long time. He warns against too easily allowing a disabled man to slip into the massage department and remain there, and gives useful details of suitable, simple gymnastic apparatus and the use of it. The publishers may be congratulated on the excellent manner in which the book has been turned out.

**The Discharged Consumptive Soldier.** By Thomas Denman, Clerk to the Brighton Insurance Committee. With a Foreword by H. de C. Woodcock, Esq., M.D. Pp. 40. Paper covers. (London: John Bale, Sons and Danielsson, Ltd. 1918. Price 1s.)

A long time ago we reviewed a French proposal for a consumptives' colony called *Le Sanatorium Village*. This is one of several similar proposals, stimulated by the large number of war consumptives to be cared for. Time will show what in this direction is practicable. Meanwhile it might be urged that with a 50 per cent. income tax very probable in the near future, more economical ideas should rule. With many religious sects contemplating union on account of their half-empty and insolvent chapels, what is the use of proposing building fresh churches for these colonies, which are mainly for the arrested cases of disease? Swimming-baths are also a luxury which could be dispensed with.

**First Aid for Foot Troubles.** By Ernest G. V. Bunting. (London: The Scientific Press, Ltd. Price 1s. 3d. net.)

This little book is practical. It contains many useful hints. In the first part the author aims to help those that have to teach, and evidently has experienced the difficulties the teacher of medical and scientific subjects is faced with when trying to instruct men whose elementary education has not been sufficient to enable them easily to acquire such knowledge. The instructor of battalion chiropodists will find advantage in reading the book.

The directions for treatment are good. In the next edition they might be made more, and in it more lavish use of such headings and display as readily catch the eye will improve the booklet and increase its attractiveness.

**Patriotism and Plenty.** By the Honble. Mrs. Lionel Guest. (London: John Lane. Price 1s.)

This book, which is labelled "A Cook-book for War Time and All Time," contains more sound sense than many a more expensive and ambitious manual. Not only is it full of excellent and rather uncommon recipes, it also holds many words of wisdom which should save the cook and the caterer from snares. The chapter on cooking "weeds" is worthy of all attention. We note many good dishes particularly suited for convalescent men, such as meat loaf, fish chowder, sausage and potato roll, scallop of corned beef and cabbage; also some good methods, little known in this country, of using potatoes. We can confidently recommend Mrs. Lionel Guest's shillingsworth to all who are seeking variety and succulence at small cost.

